# Highlight: Contribution of Ultra-Small Microbes to Global Carbon Cycles

**DOI:** 10.1093/gbe/evz089

**Published:** 2019-05-14

**Authors:** Casey McGrath

In the last five years, scientists have discovered a staggering number of ultra-small microbes, doubling the number of known lineages. Because of their extremely small size, these nanoorganisms are thought to have reduced genomes and to lack the proteins needed to carry out more complex metabolic processes. As reported in the April issue of Genome Biology and Evolution (Lannes et al. 2019), however, researchers at Sorbonne University and The Open University show that some ultra-small microbes do indeed participate in complex metabolisms and make greater contributions to global carbon cycles than previously realized.

All life on Earth is ultimately sustained by organisms such as plants and bacteria that convert carbon dioxide into organic forms in a process known as carbon fixation. While carbon fixation during photosynthesis is the most well-known of these mechanisms, there are a total of six carbon fixation pathways in nature, which differ based on the chemical reactions that occur and the enzymes used to produce these reactions. Using metagenomic sequence data collected aboard the schooner *Tara* as it crisscrossed the globe as part of the Tara Oceans project (https://oceans.taraexpeditions.org/; last accessed April 25, 2019), Eric Bapteste and study co-authors Romain Lannes, Karen Olsson-Francis, and Philippe Lopez set out to identify “novel ultra-small prokaryotes with unusual biology,” including those that may be key players in global climate cycles.

After identifying sequences in environmental samples that were likely to come from ultra-small microbes, the researchers found over 20,000 genes involved in carbon fixation. This means that, contrary to prior speculations, such nanoorganisms are likely to make significant contributions to global carbon cycling and food chains, processes that may need to be reevaluated based on this result. Commenting on the study, Karthik Anantharaman, a researcher at the University of Wisconsin who studies microbial metabolisms and their relationship to global ecosystem dynamics (Anantharaman et al. 2018) and who was not involved in the study, notes “Considering that we did not even know about the existence of such organisms, this may prompt a fundamental revisit of marine microbiology and incorporation of ultra-small microorganisms and their roles in ocean carbon cycle models.”

Notably, carbon fixation genes were found not only in microbial lineages known to include nanolineages but also in some microbial groups not previously known to harbor such miniscule members. Thus, according to Bapteste, “this way of life (being very reduced in size) is possibly more broadly distributed than currently assumed, with an underappreciated diversity of super tiny cells in the oceans.” Moreover, Anantharaman notes that the study also raises questions regarding “the involvement of these microorganisms in other biogeochemical cycles, such as nitrogen and sulfur, that are critical to the health of the oceans.”

Despite the many uncertainties, Anantharaman believes “this is a fantastic first study that sets the stage for future investigations associated with next-generation -omics approaches like genome-resolved metagenomics, transcriptomics, proteomics, and metabolomics of ultra-small organisms to understand their role in carbon fixation.” For Bapteste, future research will focus on assembling individual genomes of ultra-small microbes and determining whether the cells hosting these carbon fixation genes are self-sustaining microbes or members of larger metabolic collectives, in which different cells contribute different enzymes to the carbon fixation process. Bapteste also notes that his team’s work on nanoorganisms “is only a contribution to a larger effort to better unravel the unusual biology and diversity of environmental microbes.”
